# Miasm

**Published:** 1861-09

**Authors:** 


### HALL'S JOURNAL OF HEALTH.

**Our Legitimate Scope is almost boundless: for whatever begets pleasurable and harmless feelings, promotes Health; and whatever induces disagreeable sensations, engenders Disease.**

WE AIM TO SHOW HOW DISEASE MAY BE AVOIDED, AND THAT IT IS BEST, WHEN SICKNESS COMES, TO TAKE NO MEDICINE WITHOUT CONSULTING A PHYSICIAN.

Vol. VIII.] SEPTEMBER, 1861. [No. 9.

### MIASM.

ON the wings of the viewless winds in September, the sickliest month of the year, there is wafted an agency of disease and death, so ethereal in its nature, so intangible to mortal sense, so insinuating, so all-pervading, that no alembic can detect its presence, no prison-bar or palace-gate can prevent its entrance. It is called "MIASM;" it is an emanation from the surface of the earth wherever there is vegetation, moisture, and heat equal to eighty degrees, and is the fruitful cause of many diseases which ravage whole communities at a time, such as agues, fevers, diarrhea, dysentery, cholera, pestilence, and plague. But its laws are known, (see September JOURNAL, 1860,) and its destructive agencies can be averted by avoiding exposure and fatigue in the out-door air for the hours including sunrise and sunset, at which times a hot breakfast and supper should be eaten, by a good fire, in all prairie, flat, water-course, and lake and sea-shore situations. If the common people could only be induced to take these simple, easy, practicable, and comprehensible precautions, these diseases would be prevented as epidemics, or ~~averted~~ arrested in their progress, as certainly as that care can prevent the firing of a town, and that water will put it out. These are the teachings of science, and experiment has demonstrated their truth beyond a cavil. Yet who will take these precautions?

