# Correction

**Published:** 1861-09

**Authors:** 


### NOTICES FOR SEPTEMBER.

As the proof-reader was nodding, will the reader please wake up, and correct the nonsense, and read on first page, fifth line from the bottom, "arrested"?

